# CMR-derived myocardial strain analysis differentiates ischemic and dilated cardiomyopathy—a propensity score-matched study

**DOI:** 10.1007/s10554-021-02469-9

**Published:** 2021-11-28

**Authors:** Julia Vietheer, Lena Lehmann, Claudia Unbehaun, Ulrich Fischer-Rasokat, Jan Sebastian Wolter, Steffen Kriechbaum, Maren Weferling, Beatrice von Jeinsen, Andreas Hain, Christoph Liebetrau, Christian W. Hamm, Till Keller, Andreas Rolf

**Affiliations:** 1 Department of Cardiology, Kerckhoff Heart and Thorax Center, Benekestrasse 2-8, 61231 Bad Nauheim, Germany; 2 Medical Clinic I, Justus-Liebig-University Giessen, Giessen, Germany; 3 DZHK (German Centre for Cardiovascular Research), partner site Rhine-Main, Frankfurt am Main, Germany

**Keywords:** CMR, Feature tracking, Strain, Heart failure

## Abstract

Left ventricular (LV) longitudinal, circumferential, and radial motion can be measured using feature tracking of cardiac magnetic resonance (CMR) images. The aim of our study was to detect differences in LV mechanics between patients with dilated cardiomyopathy (DCM) and ischemic cardiomyopathy (ICM) who were matched using a propensity score-based model. Between April 2017 and October 2019, 1224 patients were included in our CMR registry, among them 141 with ICM and 77 with DCM. Propensity score matching was used to pair patients based on their indexed end-diastolic volume (EDVi), ejection fraction (EF), and septal T1 relaxation time (psmatch2 module L Feature tracking provided six parameters for global longitudinal, circumferential, and radial strain with corresponding strain rates in each group. Strain parameters were compared between matched pairs of ICM and DCM patients using paired t tests. Propensity score matching yielded 72 patients in each group (DCM mean age 58.6 ± 11.6 years, 15 females; ICM mean age 62.6 ± 13.2 years, 11 females, p = 0.084 and 0.44 respectively; LV-EF 32.2 ± 13.5% vs. 33.8 ± 12.1%, p = 0.356; EDVi 127.2 ± 30.7 ml/m^2^ vs. 121.1 ± 41.8 ml/m^2^, p = 0.251; native T1 values 1165 ± 58 ms vs. 1167 ± 70 ms, p = 0.862). There was no difference in global longitudinal strain between DCM and ICM patients (− 10.9 ± 5.5% vs. − 11.2 ± 4.7%, p = 0.72), whereas in DCM patients there was a significant reduction in global circumferential strain (− 10.0 ± 4.5% vs. − 12.2 ± 4.7%, p = 0.002) and radial strain (17.1 ± 8.51 vs. 21.2 ± 9.7%, p = 0.039). Our data suggest that ICM and DCM patients have inherently different myocardial mechanics, even if phenotypes are similar. Our data show that GCS is significantly more impaired in DCM patients. This feature may help in more thoroughly characterizing cardiomyopathy patients.

## Introduction

Heart failure with reduced ejection fraction is one of the leading causes of hospitalization, morbidity, and mortality in industrialized nations [1]. The most common aetiologies of heart failure are ischemic (ICM) and dilative (DCM) cardiomyopathies [1–3]. Although they have the same phenotype, structural myocardial changes underlying the reduced ejection fraction (EF) are different. Ischemic burden primarily affects the subendocardial compartment of the myocardium, only transmural infarction leads to a fibrotic replacement of the whole myocardial wall [4, 5]. In contrast, DCM affects endocardial as well as epicardial compartments [6].

Left ventricular ejection fraction (LV-EF) is known as a reproducible and easily assessed parameter that has prognostic value for many cardiac diseases. Nevertheless, LV-EF is a crude parameter of LV function and does not reflect LV mechanics of myocardial fibres in depth [7, 8]. Furthermore, it has been shown that myocardial fibre function can be impaired while global LV function is still normal [8].

Torrent-Guasp developed the concept of the cardiac band, which is helically folded to form different layers of myofibres, that are orientated in oblique angles to each other [9]. The advent of modern imaging techniques like diffusion tensor imaging or micro-CT has further elucidated the concept in vivo and shown that myocyte aggregates are organised in a mesh-like structure, supporting the idea of oblique angles within the mesh [10]. For the benefit of an easier understanding of these concepts we will use a simplified model in this paper, which focuses on longitudinally orientated fibres in the subendocardial layer and oblique and circumferentially orientated fibres in the subepicardial layer of the myocardium. This notion is in line with other articles focusing on endocardial and epicardial contraction [11, 12]. In this model subendocardial fibres largely contribute to longitudinal shortening while subepicardial fibres effect circumferential shortening and rotation of the myocardium.

Assessment of strain parameters by cardiac magnetic resonance imaging (CMR) offers the possibility to interrogate these different functional units separately. Global longitudinal strain (GLS) reflects subendocardial myofibre function, and global circumferential strain (GCS) reflects subepicardial myofibre function, while global radial strain (GRS) is a composite function of both GLS and GCS [11, 13, 14]. EF is a result of both GLS and GCS, and, in fact, Pedrizetti et al. showed that EF can be expressed as a function of GLS and GCS [15]. Interestingly, a given EF can be expressed by different combinations of GLS and GCS, with GCS often compensating for an early loss of longitudinal function.

It is thus possible to differentiate the extent by which each myocardial compartment is affected by heart failure. In addition, reduction in the motion of both subendocardial and subepicardial myofibres might cause more pronounced limitation of functional capacity. This, in turn, might also affect long-term prognosis.

Foley et al. reported data from a subpopulation of the VINDICATE study in which they found, that subepicardial fibres were more severely impaired in DCM patients [16]. However Foley et al. did not perform propensity score matching of ischemic and non-ischemic heart failure patients and might thus have biased groups.

Therefore, we hypothesized that the three strain parameters GLS, GCS, and GRS are well suited to detect differences in layer-specific myocardial function between ICM and DCM patients with similar EF and ventricular geometry. To avoid potential confounders due to patients being in different stages of ventricular remodelling, we performed propensity score matching to assure that both groups had similar phenotypes. In addition, the results for all heart failure patients were compared with those of healthy volunteers.

## Methods

### Patient registry

Approximately 2000 clinically indicated CMRs are performed annually at the Kerckhoff Heart and Thorax Center. All patients who are willing to take part in dedicated clinical interviews and follow-up phone calls and to supply a blood sample for our biobank are prospectively included in the BioCVI MR registry. Contraindications for CMR were incompatible metallic implants, known intolerance to gadolinium, and claustrophobia.

Additionally, to compare patient’s measures to healthy controls, we included a volunteer cohort of 64 individuals, all personnel or PhD students at our institution.

Clinical indications for CMRs were ischemia testing, reduced EF without known etiology, cardiomyopathies and myocardial inflammation.

All patients gave written informed consent. The registry was approved by the ethics committee of the University of Giessen and complies with the Declaration of Helsinki.

### Patient selection and inclusion/exclusion criteria

We identified patients from the registry with reduced EF and a scar pattern typical of coronary artery disease [subendocardial or transmural late gadolinium enhancement (LGE) corresponding to a coronary supply area] as well as patients with reduced EF without ischemic LGE.

ICM was defined by the presence of reduced EF (≤ 50%), a typical subendocardial or transmural scar in LGE or history of percutaneous coronary intervention (PCI) or coronary artery bypass grafting. DCM was identified in patients with dilated LV [indexed end-diastolic volume (EDVi) > 105 ml/m^2^], reduced EF (≤ 50%), the absence of detectable LGE consistent with inflammatory or ischemic origin, and facultatively a longitudinal or patchy intramyocardial septal midwall LGE not corresponding to a supply area of a coronary artery. Patients who were stratified as having DCM according to these criteria but having a history of CABG or PCI were excluded.

In addition, patients fulfilling the abovementioned criteria but showing active perfusion defects (beyond those due to any possible scar) were excluded from the analysis because of the influence of ischemia on strain parameters. Stress testing was performed only with clinical indication. Also patients with T2 values above the normal limit were excluded to avoid confounding by chronic inflammation.

### CMR acquisition

All subjects were examined on a 3.0 T MR scanner (Skyra, Siemens Healthineers, Erlangen, Germany) in the head-first, supine position using an 18-channel phased array surface coil.

Patients and Volunteers were examined using the same protocol albeit skipping the gadolinium injection and post contrast sequences with Late enhancement and post contrast T1.

All patients, even those with history of atrial fibrillation, were scanned in sinus rhythm to assure sufficient CINE quality for strain evaluation.

Steady-state free precession (SSFP) cine CMR sequences were acquired with a retrospective ECG-gated breath-hold technique (end-expiratory) in 2-, 3-, and 4-chamber long-axis views as well as 13 short-axis slices from base to apex. Typical parameters were echo time (TE) 1.38 ms, repetition time (TR) 3.1 ms, flip angle 55°, bandwidth 962 Hz/px, field of view (FOV) 380 mm, voxel size 1.8 × 1.8 × 8.0 mm, interslice gap 2 mm, and temporal resolution 30 ms. Volumetric measurements were performed by using cvi42 software (circle cardiovascular imaging, Calgary, Canada). Endocardial and epicardial contours were drawn on the end-diastolic and end-systolic LV borders excluding trabeculations.

We also included patients undergoing stress CMR protocols. In those CINE imaging was performed about 4 min after injection of 400 ug regadenosone.

### T1 mapping

Native T1 maps were generated by using modified look locker sequences (MOLLI 3(2)3(2)5, 50° Flip Angle, Goethe CVI®, Calgary Canada) at the LV base, midventricular, and apical portions. Typical parameters were TE 1.14 ms, TR 2.8 ms, bandwidth 1085 Hz/px, voxel size 1.4 × 1.4 × 8.0 mm, slice thickness 8 mm, non-selective inversion pulse, and ECG-gated antegrade SSFP single-shot read out. Mean native T1 values were calculated in a region of interest in the midventricular septum that was defined by an experienced examiner. To determine the global T1 time, ROIs were drawn manually in the midventricular septal wall according to the ConSept study as published previously [17].

### Late gadolinium enhancement

Inversion recovery segmented gradient echo sequences were acquired 10 to 15 min after intravenous injection of Dotarem® (0.15 mmol/kg bodyweight) in 13 short-axis and 2-, 3-, and 4-chamber long-axis views. Typical parameters were TE 1.97 ms, TR 5.0 ms, flip angle 20°, bandwidth 289 Hz/px, voxel size 1.4 × 1.4 × 8.0 mm, and slice thickness 8 mm, with TI tailored to efficiently nullify the myocardial signal. LGE was visually evaluated using a 16-segment model. LGE expansion of more than 70% of the wall was defined as transmural.

### Inter- and Intraobservervariabiltiy

Datasets of 30 patients were randomly selected out of the whole cohort and analyzed twice by JV and AR to compute inter- and intraobservervariability.

### Feature tracking

**Fig. 1:**
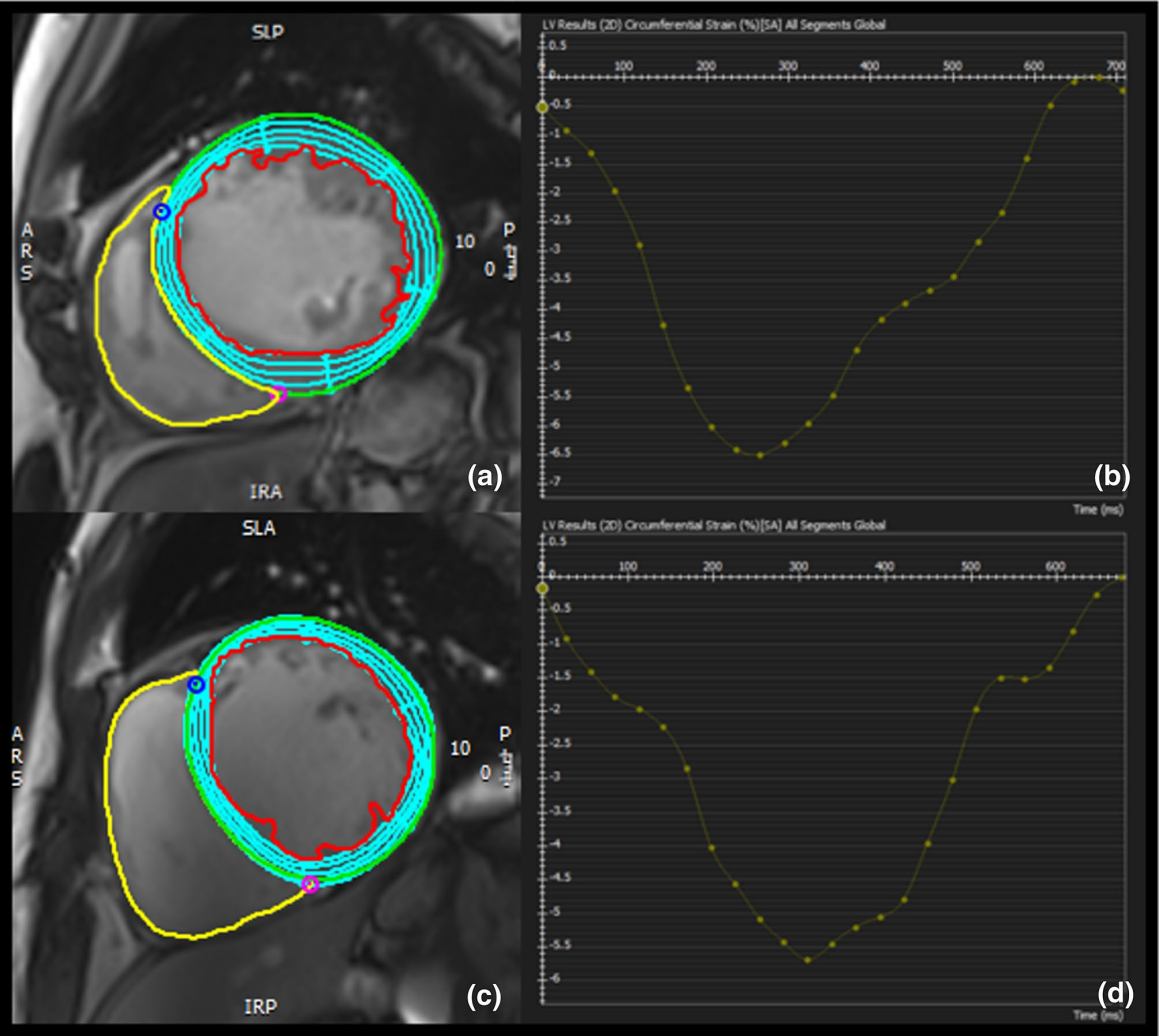
Example of the calculation of GCS in an ICM patient (**a**, **b**) and a matched DCM patient (**c**, **d**) by cvi42 software (circle cardiovascular imaging, Calgary, Canada). *DCM* dilated cardiomyopathy, *GCS* global circumferential strain, *ICM* ischemic heart disease

2D-LV strain and systolic strain rates were calculated using the feature tracking (FT) module of cvi42 (Fig. 1). The LV endocardial and epicardial borders were traced at the end-diastolic phase. LV trabeculations were carefully excluded. The baseplane was defined on 4-chamber long axis views. The software automatically propagated contours throughout all phases and derived global strain and systolic strain rates in longitudinal, circumferential, and radial directions. The propagated myocardial tissue across the cardiac cycle was verified by the operator to ensure the accuracy of the propagation. GCS, global systolic circumferential strain rate (sysGCSR), GRS, and global systolic radial strain rate (sysGRSR) were obtained using short-axis cine views. GLS and global systolic longitudinal strain rate (sysGLSR) were obtained from 2- and 4-chamber long-axis views. If propagation was not plausible, endocardial contours were slightly shifted to enable different feature recognition, which might improve strain measurement. Only good quality strain data are entered into the database, therefore all patients extracted had proper image quality.

### Statistics

Data are presented as mean ± standard deviation. Patients were matched using a propensity score model controlling for LV-EF, LV-EDVi, and native T1 with one-to-one nearest-neighbour matching and a calliper of 0.1 (STATA psmatch2 module Leuven and Sianesi [18]). Mean values between groups were compared using Student’s t test for paired data. Differences in frequencies were compared using chi-squared tests. A p value of less than 0.05 was considered to indicate statistical significance. We computed Lin’s Rho concordance index to measure inter- and intraobservervariability. All tests were computed using Stata17 (StataCorp, College Station, Texas, USA).

## Results

### Patient characteristics

**Fig. 2:**
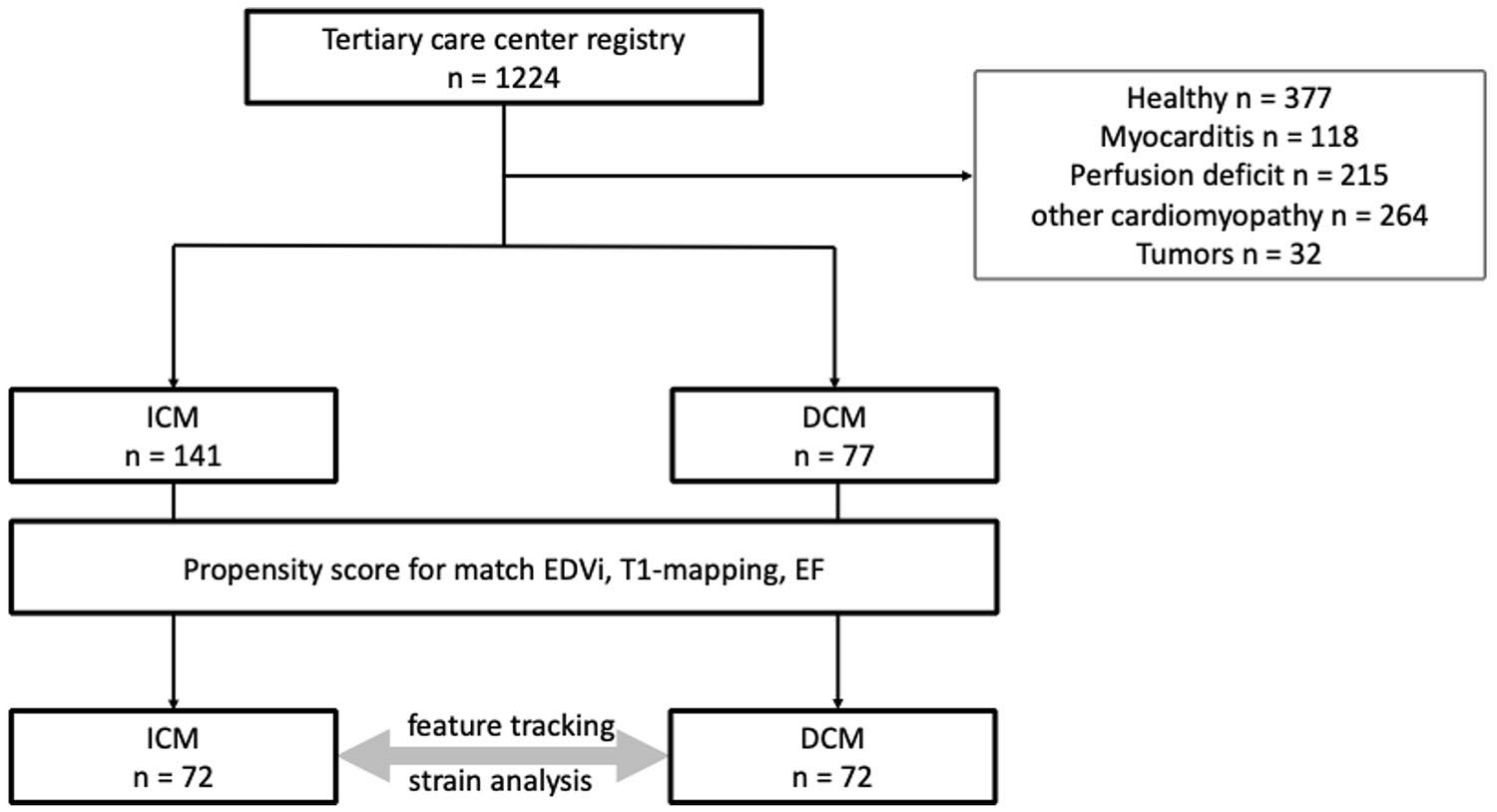
Flow chart for patient selection. Out of our tertiary care centre registry with 1224 patients we identified 141 patients with ICM (defined by typical subendocardial or transmural LGE) and 77 patients with DCM (reduced EF, absence of LGE or midwall sign). After matching for EF, T1 mapping, and EDVi, 72 patients remained in each group. *DCM* dilated cardiomyopathy, *EDVi* indexed end-diastolic volume, *EF* ejection fraction *ICM* ischemic cardiomyopathy

From April 2017 to October 2019 1224 patients and 64 healthy volunteers were included in the registry. Among these patients we identified 77 with DCM and 141 with ICM according to the criteria defined above. After propensity score matching, 72 well-matched patients in each group with DCM and ICM were selected (Fig. 2). In the DCM group the mean age was 58.6 ± 11.5 years and 11 patients (15%) were female. In the ICM group the mean age was 62.6 ± 13.2 years and 20 patients (28%) were female (Table 1). Further baseline characteristics are given in Table 1. The 64 healthy volunteers included were younger than heart failure patients, had a larger proportion of female volunteers than the patient groups, a lower body mass index, and significant differences in all CMR parameters (Table 3).

**Fig. 3:**
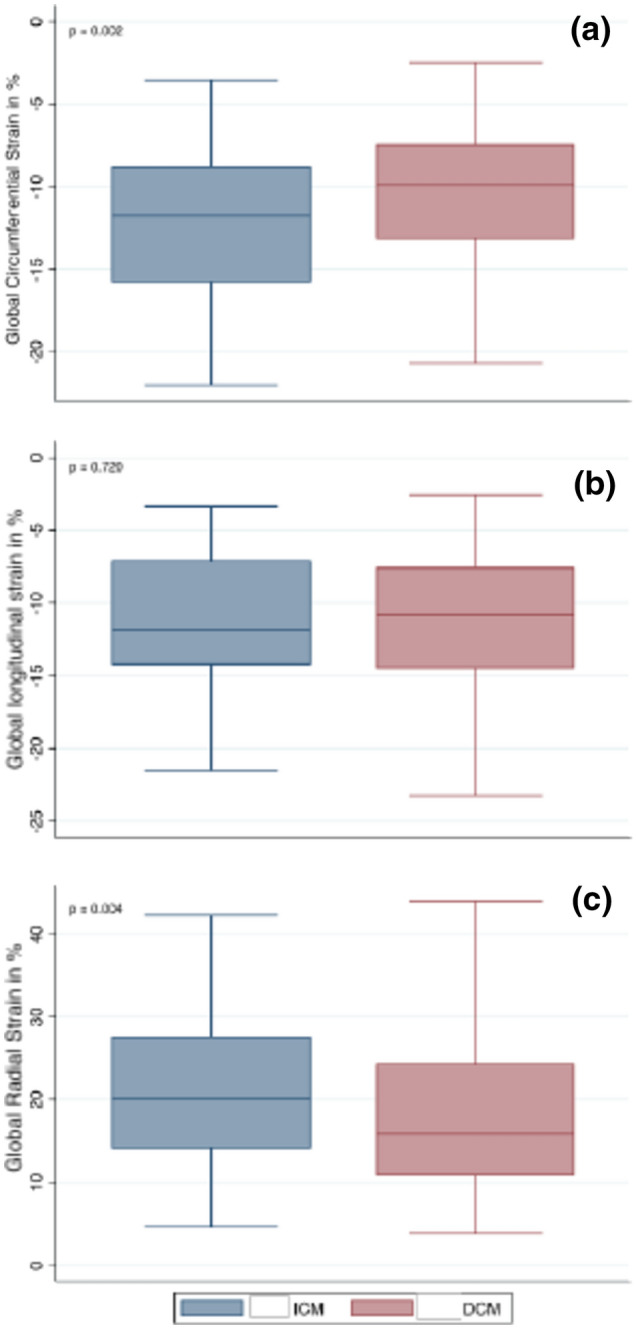
Box-and-whisker plots comparing DCM and ICM patients for **a** GCS, **b** GLS, and **c** GRS. DCM dilated cardiomyopathy, ICM ischemic cardiomyopathy, *GCS* global circumferential strain, *GLS* global longitudinal strain, *GRS* global radial strain

**Table 1:** Baseline characteristics of DCM and ICM patients

Value	DCM, n = 72 mean ± SD or n (%)	ICM, n = 72 mean ± SD or n (%)	p
Age (y)	58.6 ± 11.5	62.5 ± 13.2	0.084
Female	11 (15)	20 (28)	0.44
BMI (kg/m^2^)	28.4 ± 4.9	28.6 ± 6.0	0.8
NT-Pro-BNP (ng/L)	2690 ± 5063	2870 ± 2869	0.69
Troponin T (ng/L)	49.4 ± 119	114.5 ± 230	0.071
Serum creatinine (mg/dl)	0.98 ± 0.9	1.05 ± 0.49	0.58
NYHA III	16 (22)	11 (15)	0.85
NYHA IV	14 (19)	9 (13)	
Atrial fibrillation	18 (25)	20 (28)	0.712
Diabetes	8 (11)	21 (29)	0.635
Prior CABG	0 (0)	15 (21)	n.a
Prior PCI	6 (8)	29 (40)	0.72

*BMI* body mass index, *CABG* coronary artery bypass graft, *DCM* idiopathic dilated cardiomyopathy, *ICM* ischemic heart disease, *NT-Pro-BNP* N-terminal pro-brain natriuretic peptide, *NYHA* New York heart association, *PCI* percutaneous coronary intervention

### Quality of propensity score matching

To assure that propensity score matching reduced any possible bias between DCM and ICM patients, we computed standardized differences between the matching parameters before and after matching. There was significant bias in both groups regarding LV-EF and LVEDVi before matching, which became almost negligible after matching.

LVEF before matching DCM 32.2% vs ICM 43.1% bias − 74.6% p = 0.001.

LVEF after matching DCM 32.2% vs ICM 30.9% bias 9.0, bias reduction 87%

LVEDV before matching DCM 127.2 ml vs ICM 103 ml bias 69.2% p = 0.0001.

LVEDV after matching DCM 127.2 ml vs ICM 128 ml bias − 2.2%, bias reduction 96.9%

T1 native before matching DCM 1165.7 vs. ICM 1167.5 bias − 2.6 p = 0.87.

T1 native after matching DCM 1165.7 vs. ICM 1179.3 bias 20.2, bias reduction − 13%, p = 0.21.

### CMR measurements

There was no difference between DCM and ICM patients in the matched parameters LV-EF (p = 0.36), EDVi (p = 0.251), native T1 values (p = 0.86) and ECV (p = 0.9) (Table 2). Furthermore, no difference was found for the remaining volumetric measurements end-systolic volume (ESVi) (p = 0.26) and LV mass i (p = 0.52).

**Table 2:** CMR measurements in matched DCM and ICM patients

Parameter	DCM, n = 72 MV ± SD	ICM n = 72 MV ± SD	p
LV-EDVi (ml/m^2^)	127.2 ± 30.1	121.1 ± 41.8	0.2506
LV-ESVi (ml)	88 ± 4	82.4 ± 4.5	0.26
LV-EF (%)	32.2 ± 13.5	33.8 ± 12.1	0.3556
LV mass i (g)	63.9 ± 2.2	61.7 ± 2.7	0.5221
T1 mapping (ms)	1165.7 ± 58.5	1167.8 ± 69.7	0.8620
ECV%	0.27 ± .006	0.27 ± .009	0.9064
GRS (%)	17.1 ± 8.5	21.2 ± 9.7	0.0041
GCS (%)	− 10.0 ± 4.5	− 12.2 ± 4.8	0.0018
GLS (%)	− 10.9 ± 5,4	− 11.2 ± 4.7	0.7200
SysGRSR (1/s)	0.88 ± 0.42	1.05 ± 4.7	0.0277
SysGCSR (1/s)	− 0.58 ± 0.28	− 0.59 ± 0.34	0.8828
SysGLSR (1/s)	− 0.63 ± 0.18	− 0.48 ± 0.50	0.0332

*DCM* idiopathic dilated cardiomyopathy, *EDVi* indexed end-diastolic volume, *EF* ejection fraction, *ESV* end-systolic volume, *GCS* global circumferential strain, *GFR* glomerular filtration rate, *GLS* global longitudinal strain, *GRS* global radial strain, *ICM* ischemic heart disease, *LV* left ventricle, *sysGCSR* systolic global circumferential strain rate, *sysGLSR* systolic global longitudinal strain rate, *sysGRSR* systolic global radial strain rate

### Late gadolinium enhancement

In the ICM group 47 patients had at least one segment showing transmural LGE, and 25 patients had only subendocardial LGE. In the DCM group 27 patients had intramural LGE. ICM patients had significantly more segments showing LGE than DCM patients (5.8 ± 3.8 vs. 1.6 ± 2.1; p = 0.0001).

### Strain analysis

We found excellent inter-rater-reproducibility for GCS, GLS and GRS and good inter-rater-reproducibility for the respective strain rates (Lin’s rho-C: GLS 0.969, p = 0.0001; GCS 0.931, p = 0.0001; GRS 0.955, p = 0.0001; GLSR 0.893 p = 0.0001; GCSR 0.891, p = 0.0001, GRSR 0.942; p = 0.0001). The same holds true for intra-rater-reproducibility (GLS 0.979, p = 0.0001; GCS 0.981, p = 0.0001; GRS 0.988, p = 0.0001; GLSR 0.905, p = 0.0001; GCSR 0.909, p = 0.0001; GRSR 0.954, p = 0.0001).

All strain parameters were lower in patients with heart failure than in our cohort of 64 heathy volunteers (Table 3). No difference was found in GLS in DCM vs. ICM patients (− 10.9 ± 5.4% vs. − 11.2 ± 4.7%, p = 0.720), whereas GCS and GRS were lower in DCM patients (− 10 ± 4.5% vs. − 12.2 ± 4.8%, p = 0.002 and 17.1 ± 8.5 vs. 21.2 ± 9.7%, p = 0.039) (Table 2, Fig. 3). Furthermore, DCM patients showed a higher sysGLSR (p = 0.033), whereas sysGRSR (p = 0.028) was lower (Table 2).

**Table 3:** Volumetric measurements and strain parameters in healthy volunteers and patients with heart failure

Parameter	Healthy volunteer n = 64 MV ± SD	Heart failure n = 208 MV ± SD	p
LV-EDVi (ml/m^2^)	86.3 ± 12.2	111.7 ± 37.9	0.00001
LV-ESVi (ml)	31.8 ± 5.5	71.9 ± 38.5	0.00001
LV-EF (%)	63.2 ± 4.7	39.1 ± 15.6	0.00001
LV mass i (g)	38.9 ± 15.9	58 ± 20.3	0.00001
T1 mapping (ms)	1122 ± 28	1166.75 ± 64	0.00001
GRS (%)	46.7 ± 8.7	23.8 ± 12.7	0.00001
GCS (%)	− 15.2 ± 6.8	− 13.2 ± 5.7	0.00001
GLS (%)	− 20.8 ± 2.5	− 12.6 ± 5.4	0.00001
SysGRSR (1/s)	2.45 ± 0.58	1.17 ± 0.61	0.00001
SysGCSR (1/s)	− 1.15 ± 0.18	− 0.65 ± 0.37	0.00001
SysGLSR (1/s)	− 1.06 ± 0.21	− 0.59 ± 0.42	0.00001

*EDVi* indexed end-diastolic volume, *EF* ejection fraction, *GCS* global circumferential strain, *GLS* global longitudinal strain, *GRS* global radial strain, *LV* left ventricle, *sysGCSR* systolic global circumferential strain rate, *sysGLSR* systolic global longitudinal strain rate, *sysGRSR* systolic global radial strain rate

If the strain analysis was restricted to those patients exhibiting at least one transmural segment of LGE (47 patients), GCS was still found to be lower in DCM patients by almost the same amount as in the complete group (− 9.6 ± 4.5% vs. − 12 ± 4.8%, p = 0.007). However, in an analysis of covariance of GCS between the groups, with the number of transmural segments as covariate, the difference becomes insignificant.

We also divided the patients into two groups according to the median EF, which was 32.2%. While GCS of DCM patients was significantly lower in the group of patients with EF below the median (− 7% ± 0.6% vs 10.6% ± 0.8%p = 0.0019), this difference could not be observed above the median EF (− 13.4% ± % 14.2% ± %, p = 0.36).

## Discussion

We examined whether different CMR-derived strain parameters can detect differences in layer-specific myocardial function between ICM and DCM patients with similar EF and ventricular geometry.

The main findings of our study were that:

i. all heart failure patients showed significantly reduced strain values compared with those of healthy controls, and
ii. DCM patients had lower GCS and GRS than patients with ICM, whereas GLS was reduced similarly in the two groups. This effect was especially driven by patients with ejection fractions below the median.

These findings suggest that DCM affects all layers of the myocardium similarly, whereas in ICM patients dysfunctional fibres are located primarily in the subendocardium. Hence, we found that GCS can be used to differentiate the two entities.

The results were consistent between strain and strain rates, which is important as strain rates are at the one hand considered to be even more subtle parameters of fibre function but have at the other hand lower reproducibility. So the agreement between strain and strain rates supports the validity of our findings.

Similar results were reported for a subgroup analysis of the VINDICATE cohort in ischemic and non-ischemic cardiomyopathies in which strain analysis was performed by using tagging [16]. While there was no significant difference in GLS, twist and torsion were significantly reduced in non-ischemic cardiomyopathies in this study. This is in good agreement with our findings, as twist and torsion are also attributed to epicardial layers [16].

Our findings may be explained by two principle mechanisms involving, first, the fibre architectural arrangement in the different myocardial layers and, second, the different pattern of myocardial damage and fibrosis that dominates in either DCM or ICM. To achieve the optimal myocardial contraction, the healthy LV is arranged in myocardial layers with different meshed fibre orientations. The subepicardial layer has a left-handed 60° fibre orientation that generates a rotational motion from base to the apex, whereas the subendocardial layer consists of right-handed 80° longitudinal fibres that cause longitudinal shortening [19, 20]. As apex and base rotate in opposite directions, a twisting of the left ventricle occurs. Both twisting and longitudinal shortening cause a torsional motion of the ventricle, which in turn causes a reduction of the ventricular volume from EDV to ESV that is described by the EF.

From this, one can conclude that aetiologies affecting primarily subendocardial fibres will predominantly influence longitudinal shortening, and that aetiologies affecting all myocardial layers will influence both longitudinal shortening as well as circumferential strain, twist, and torsion. EF is a summation of both the circumferential and longitudinal movement. Therefore, using EF as a single functional parameter is not sufficient to describe myocardial mechanics in depth [7, 21, 22].

Strain analysis yields three parameters that account for myocardial thickening and shortening in three directions independently: while GLS represents a shortening from base to apex, radial and circumferential strain reflect the concentric thickening and rotational motion [13, 23]. Indeed, strain analysis can detect subtle myocardial damage before EF is reduced [8, 24, 25]. Various echocardiographic and CMR-based studies investigated the diagnostic accuracy and prognostic value of strain analysis [13, 26–28]. Speckle tracking, the main echocardiographic technique used for strain analysis, is limited due to image quality and anatomical variations [27]. CMR FT provides the possibility to analyse standard SSFP cine sequences retrospectively with image quality that is superior to that of echocardiography. No additional sequence is needed as is necessary in tagging, which was used for the VINDICATE study [13, 16]. This offers deep insight into myocardial mechanics in routine diagnostics without additional costs.

Strain parameters, however, are known to be correlated with EF and fibrosis [29–32]. Therefore, we tried to control for these effects by using propensity score matching between ICM and DCM patients. We generated two groups of patients that had very similar values of EF and EDVi and also degree of fibrosis, as represented by similar T1 relaxation times. Despite the similar phenotypes of DCM and ICM patients, our study confirmed that these cardiomyopathies have inherently different mechanics.

Hypoxemia triggers necrosis of myocytes that follows a wavefront phenomenon: it begins at the subendocardial layer and spreads towards the epicardium, with its extent being dependent on the duration of ischemia [5]. A regional scar consisting of replacement fibrosis develops that can be made visible by LGE imaging. Long-term ischemia due to ST elevation myocardial infarction leads to complete destruction of all myocardial layers, including the subepicardium. In subendocardial infarction the loss of predominantly endocardial fibres is compensated by hypertrophy of the epicardial fibres [4, 5, 33], which leads to an increased GCS that compensates for the loss of longitudinal function [34].

That there is a regional reduction in strain parameter values after infarction is already known, but strain analysis can even detect transient ischemia during dobutamine stress CMR [33, 35]. On the other hand, in DCM a globally diseased myocardium with diffuse reactive interstitial fibrosis due to increased collagen proliferation of myofibroblasts with progressive onset governs the histopathological changes [6]. In the evaluation of DCM patients by CMR, strain analysis is of special interest because global strain parameters correlate with EF and are viewed as independent risk factors beyond the traditional parameters EF and LGE [36–38]. Our data show for the first time in a propensity-matched cohort that the impact on all myocardial layers in DCM patients can be detected by significantly reduced GCS and GRS compared with the values for ICM patients, whereas EF and ventricular phenotype are not significantly different between ICM and DCM patients.

Interestingly, the GLS of DCM patients was not different from that of ICM patients, although ICM patients lost subendocardial segments due to necrosis and replacement fibrosis. Based on this pathomechanism we expected a lower GLS in ICM patients. The fact that DCM involves the complete myocardium, whereas ICM is a more segmental process, could be an explanation for the similarity between GLS values.

We also examined the group of ICM patients with transmural infarctions in which GCS could be more severely reduced and all myocardial layers are disturbed. In comparing ICM patients with at least one transmural infarcted segment with their DCM counterparts, we found almost the same effect size in GCS as in the whole group, which suggests that epicardial myofibres in remote regions compensate for the transmural fibre loss within the infarct region. However, if we include increasing numbers of transmurally infarcted segments as covariates in an ANCOVA model, the difference in GCS between the two aetiologies is diminished, which does not prove but is consistent with our hypothesis of layer-specific damage in ICM and DCM. Because with increasing numbers of transmural segments, ICM begins to resemble DCM as an increasing portion of the epicardial layers is dysfunctional.

Despite different aetiologies between DCM and ICM, increasing fibrosis is the leading pathological mechanism in ischemic and non-ischemic heart failure, leading to ventricular dysfunction, stiffness, and cardiac remodelling. Consequently, lower strain parameters can be measured in both DCM and ICM groups compared with those of healthy volunteers. While GLS is known to be reduced in early stages of cardiomyopathy, a lowering of GCS is a phenomenon in advanced disease [39, 40]. Our results confirm that in cases having the same phenotype, strain analysis is able to detect the global involvement and altered myocardium associated with DCM.

### Limitations

FT strain analysis is a widely used technique to quantify myocardial deformation by using standard SSFP cine sequences. Nevertheless, normal values are scarce in the literature, and the optimal threshold to differentiate diseased and healthy myocardium, especially for circumferential strain, has yet to be defined. Further, FT strain analysis shows inferior performance in terms of accuracy and reproducibility, especially for segmental strain analysis, compared with other strain methods like SENC and tagging [41]. Therefore, we focused on global strain parameters that have been found to have good reproducibility. DCM was defined by the absence of LGE in addition to a septal midwall sign. There was no histological confirmation of the origin of DCM patients in our study, so the potential influence of an active viral inflammation on strain analysis in these patients cannot be ruled out completely. However, we included only T2 normal patients to control for this potential bias. The proportion of women in the DCM cohort was slightly lower than one might expect, however the gender difference between both groups was not significantly different. We therefore think this underrepresentation of females in the DCM cohort will not influence the inferences made in this paper. Patients who underwent this protocol as part of their stress perfusion examination had CINE sequences after injection of regadenoson. This might slightly increase EF. However strain values are all affected in the same way, therefore regadenosone should have no effect on layerspecific contraction patterns.

## Conclusion

Despite these caveats, our data strongly suggest that CMR FT-based strain analysis is able to differentiate underlying myocardial mechanics between ICM and DCM, even if phenotypes are similar. The ability to discriminate these two conditions may aid in deeper understanding of pathophysiology in the future.

## Declarations

### Data availability

The data underlying this article cannot be shared publicly in order to protect the privacy of individuals who participated in the study. The data will be shared on reasonable request to the corresponding author.
